# Prolonged adrenal suppression after osilodrostat discontinuation in a patient with Cushing’s disease with eventual hypercortisolism relapse: Case Report and literature review

**DOI:** 10.3389/fmed.2025.1629387

**Published:** 2025-09-15

**Authors:** Lukasz Dzialach, Wioleta Respondek, Anna Siejka, Przemyslaw Witek

**Affiliations:** ^1^Department of Internal Medicine, Endocrinology and Diabetes, Medical University of Warsaw, Warsaw, Poland; ^2^Department of Internal Medicine, Endocrinology and Diabetes, Mazovian Bródnowski Hospital, Warsaw, Poland; ^3^Department of Clinical Biochemistry, The Children’s Memorial Health Institute, Warsaw, Poland

**Keywords:** adrenal insufficiency, adrenocortical blockade, Cushing’s disease, osilodrostat, recombinant human growth hormone

## Abstract

Osilodrostat is a potent oral steroidogenesis inhibitor that is an effective medical therapy in the management of patients with endogenous Cushing syndrome. However, due to its high therapeutic potential, it is associated with a high risk of inducing adrenal insufficiency (AI). Recently, it has also been reported that patients may experience prolonged adrenal suppression during osilodrostat treatment that persists despite its withdrawal. In this paper, we present a male patient with persistent Cushing’s disease (CD) who experienced several episodes of AI during long-term treatment with osilodrostat. Ultimately, due to the patient’s very low dose of osilodrostat, it was decided to discontinue the therapy after 270 weeks in total. Following the cessation of osilodrostat, the patient commenced treatment with recombinant human growth hormone due to severe growth hormone deficiency, which revealed an underlying cortisol deficiency, likely caused by a prolonged adrenocortical blockage induced by osilodrostat, requiring the initiation of hydrocortisone replacement therapy. During and after the osilodrostat therapy, we additionally observed a low serum concentration of dehydroepiandrosterone sulfate (DHEA-S) despite elevated plasma adrenocorticotrophin. This finding suggested potential inhibition of adrenal steroidogenesis upstream of 11β-hydroxylase. A urine steroid profile performed 40 weeks after discontinuing osilodrostat showed reduced or borderline excretion of cortisol metabolites, as well as significantly decreased excretion of DHEA metabolites. Finally, 62 weeks after the last exposure to osilodrostat, the patient presented with clinical and biochemical features of relapse of hypercortisolemia, and osilodrostat was reintroduced. This case highlights the importance of close monitoring in patients treated with osilodrostat, as hypocortisolemia can arise suddenly and unexpectedly at any point during treatment, even in those on stable doses. Additionally, it indicates that osilodrostat has the potential to induce prolonged adrenal blockade, even after treatment has ceased. The unexpected persistence of adrenal suppression suggests unknown long-term effects of osilodrostat that require further investigation.

## Background

Adrenocorticotrophin (ACTH)-secreting pituitary tumors are the primary cause of endogenous Cushing’s syndrome (CS) traditionally referred to as Cushing’s disease (CD). Transsphenoidal selective adenomectomy remains a preferred first-line treatment in most patients (1, 2). In cases of incomplete resection, recurrent disease, or when surgery is not feasible, contraindicated, or declined, medical therapy has emerged as a second-line option in recent years due to the availability of new pharmacological agents. These drugs target adrenal steroidogenesis, somatostatin and dopamine receptors in the pituitary gland, as well as glucocorticoid receptors (2, 3).

Osilodrostat is a potent oral inhibitor of 11β-hydroxylase (11β-OH), the enzyme that catalyzes the final step of cortisol synthesis. It provides both rapid and sustained control of cortisol production (4, 5). However, its high efficacy is associated with a relatively frequent occurrence of adrenal insufficiency (AI) episodes, as observed in both clinical trials and real-world studies (6–9). AI typically occurs during the titration phase and dose adjustments (5, 10); however, it may also develop in patients receiving a stable dose during long-term treatment (8, 9, 11). Prolonged adrenal blockade may persist even after osilodrostat discontinuation (12–16). This raises the possibility that treatment with osilodrostat can be interrupted in selected patients without an immediate recurrence of hypercortisolism.

In this paper, we present a case of a patient with persistent Cushing’s disease (CD) who experienced several episodes of hypocortisolemia during long-term treatment with osilodrostat, necessitating a gradual dose reduction and eventual discontinuation of the drug. Following withdrawal, the patient commenced recombinant human growth hormone (rhGH) therapy due to severe growth hormone deficiency. However, this treatment unmasked an underlying cortisol deficiency, requiring the initiation of hydrocortisone replacement therapy. Approximately 62 weeks after the final dose of osilodrostat, the patient exhibited clinical and biochemical signs of hypercortisolemia relapse, prompting the reintroduction of osilodrostat.

## Case presentation

In February 2013, the then 31-years-old patient was diagnosed with CD during the differential diagnosis of hypertension, which was accompanied by unintentional weight gain with central adipose tissue redistribution, purple striae on the trunk, proximal myopathy, hypokalemia, dyslipidemia, and reduced bone mineral density. Hormonal assays confirmed ACTH-dependent hypercortisolemia, and stimulation tests with CRH, desmopressin, and a high-dose dexamethasone suppression test suggested a pituitary source of ACTH hypersecretion. However, pituitary magnetic resonance imaging (MRI) did not reveal a pituitary tumor. Subsequent inferior petrosal sinus sampling (IPSS) with CRH stimulation confirmed the diagnosis of CD.

In May 2013, the patient underwent an unsuccessful transsphenoidal exploration of the sella turcica, with persistent hypercortisolemia. A second procedure was performed in November 2013; however, no pituitary tumor was detected. Based on IPSS findings, a right-sided hemihypophysectomy was performed. The disease did not remit, and the surgery led to hypopituitarism affecting the remaining pituitary axes. As a result of persistent CD, medical therapy with ketoconazole was initiated, followed by levothyroxine and testosterone replacement.

Ketoconazole treatment was only partially effective, requiring comprehensive management of comorbidities associated with hypercortisolemia. Between October 2015 and October 2016, the patient participated in the CSOM230G2304 clinical trial, receiving long-acting pasireotide, which led to partial disease control. After completion of the trial, ketoconazole was reintroduced due to the lack of available alternative treatment options.

Due to refractory CD, osilodrostat therapy was initiated in September 2018. At that time, the hormonal tests were as follows: morning serum cortisol – 638 nmol/L (reference range [RR]: 127.0–567.0 nmol/L), morning ACTH – 9.3 pmol/L (RR: 1.3–11.1 pmol/L), mean urinary free cortisol (mUFC) – 580.0 nmol/24 h (4.2 × upper limit of normal [ULN]; RR: 11.0–138.0 nmol/24 h), and mean late-night salivary cortisol (mLNSC) – 11.9 nmol/L (4.7 × ULN; RR: ≤2.5 nmol/L). The patient initially received osilodrostat at a dose of 2 mg twice daily (BID), with dose adjustments every 4 weeks, reaching 5 mg BID. By week 17, disease control had not been achieved: mUFC – 164.4 nmol/24 h (1.2 × ULN), mLNSC – 4.2 nmol/L (1.7 × ULN). At week 20, the patient reported fatigue, muscle weakness, and arthralgia. Biochemical evaluation confirmed AI: morning serum cortisol – 88 nmol/L, morning ACTH – 15.8 pmol/L. Temporary hydrocortisone replacement therapy was introduced, and the osilodrostat dose was reduced to 3 mg BID.

Over the following weeks of treatment, the patient maintained good biochemical control, with regression of the clinical features of CD. However, at week 60, he again exhibited signs of AI: morning serum cortisol of 91 nmol/L, ACTH of 15.1 pmol/L, necessitating a further reduction of the osilodrostat dose to 2 mg BID. A similar episode occurred at week 96, prompting a dose reduction to 1 mg BID.

In the following weeks, no further clinical signs of AI were observed; however, due to persistently low morning serum cortisol and mUFC levels, the osilodrostat dose was gradually tapered to 1 mg every third evening by week 156. This very low dose was maintained for 114 weeks until December 2023. After a total of 270 weeks of osilodrostat treatment, the therapy was ultimately discontinued. The patient has since been closely monitored with regular biochemical assessments for recurrence of hypercortisolemia, including periodic LNSC measurements.

In August 2024, rhGH therapy was initiated as part of the National Program for the Treatment of Severe Growth Hormone Deficiency in Adults in Poland (17). During the 1-month follow-up, the patient reported fatigue, muscle weakness, and borderline low blood pressure, raising concern for AI. Hormonal testing revealed a low morning serum cortisol level of 91 nmol/L, with a peak serum cortisol level of 317 nmol/L during the cosyntropin stimulation test, confirming the diagnosis of AI. The ACTH concentration at that time was 25.3 pmol/L. Mineralocorticoid production remained unaffected – aldosterone: 94.8 pg/mL (RR: 25.2 – 392.0 pg/mL, direct renin concentration: 22.4 μIU/mL (RR: 4.4 – 46.1 μIU/mL). Hydrocortisone replacement therapy was initiated at a dose of 15 mg per day, and the dosage of rhGH was adjusted over the following months. These interventions led to a general improvement in the patient’s well-being.

However, in February 2025, the patient showed clinical and biochemical signs of relapsed hypercortisolemia. Key findings included elevated blood pressure, a weight gain of approximately 5 kg, and deterioration of the lipid profile. Hormonal evaluation revealed the following: morning serum cortisol – 438.6 nmol/L, midnight serum cortisol – 340.1 nmol/L, morning ACTH – 15.9 pmol/L, mUFC) – 128.3 nmol/24 h, mLNSC – 5.4 nmol/L. Consequently, the patient was reintroduced to osilodrostat treatment at a low, single daily dose of 1 mg in the evening.

**FIGURE 1:**
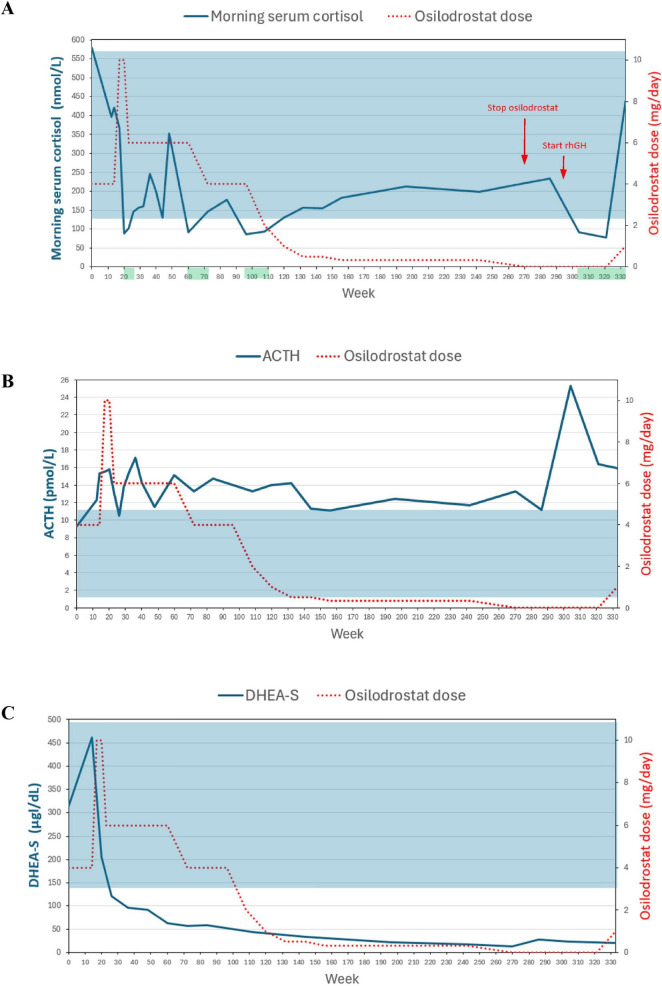
The charts show the change in morning serum cortisol **(A)**, plasma ACTH **(B)** and serum DHEA-S **(C)** concentration from the start of the osilodrostat treatment. The shaded area corresponds to the reference range of given parameter. The red arrows in chart **(A)** indicates the time when osilodrostat was discontinued and rhGH therapy was introduced. The green shading in chart **(A)** indicates the period during which the patient was receiving hydrocortisone supplementation.

It is worth mentioning the evolution of DHEA-S and ACTH concentrations observed during osilodrostat therapy. Before starting the treatment, the patient’s DHEA-S concentration was 315 μg/dL (RR: 140–492 μg/dL). As expected, the DHEA-S concentration initially increased during treatment, reaching a maximum of 461 μg/dL in the 14th week of therapy. Subsequently, a gradual decline in DHEA-S concentration was observed; from the 26th week of treatment, it fell below the RR and did not normalize during the subsequent observation period, even after osilodrostat discontinuation and hypercortisolemia relapse. The ACTH concentration, which was within the normal range at baseline, rose above the RR after the initiation of osilodrostat therapy and remained elevated throughout the follow-up. Figure 1 summarizes the serum DHEA-S and plasma ACTH concentrations since the initiation of osilodrostat treatment.

We performed the urine steroid profile in October 2024 (hydrocortisone was withdrawn 48 h prior urine collection). As expected, decreased or low-normal urinary excretion of cortisol metabolites was observed. The concentrations of mineralocorticoid metabolites were normal. Additionally, the analysis revealed low excretion of DHEA and its metabolites, while the excretion of androstenedione metabolites remained within the normal range. Despite the persistent adrenal suppression, the excretion of 11-deoxycortisol metabolites was within the reference range. The results of the urinary steroid profile are summarized in Table 1.

**TABLE 1:** Results of urine steroid profile.

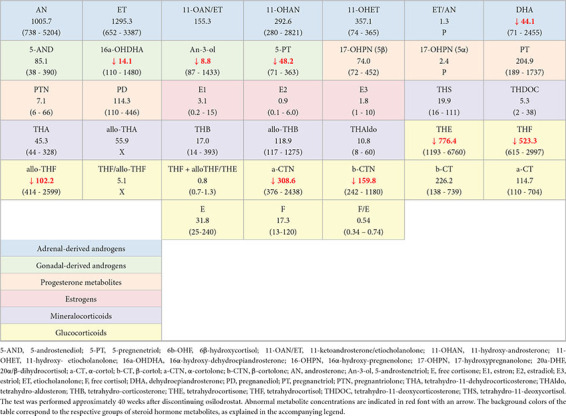

5-AND, 5-androstenediol; 5-PT, 5-pregnenetriol; 6b-OHF, 6β-hydroxycortisol; 11-OAN/ET, 11-ketoandrosterone/etiocholanolone; 11-OHAN, 11-hydroxy-androsterone; 11-OHET, 11-hydroxy- etiocholanolone; 16a-OHDHA, 16α-hydroxy-dehydroepiandrosterone; 16-OHPN, 16α-hydroxy-pregnenolone; 17-OHPN, 17-hydroxypregnanolone; 20a-DHF, 20α/β-dihydrocortisol; a-CT, α-cortol; b-CT, β-cortol; a-CTN, α-cortolone; b-CTN, β-cortolone; AN, androsterone; An-3-ol, 5-androstenetriol; E, free cortisone; E1, estron; E2, estradiol; E3, estriol; ET, etiocholanolone; F, free cortisol; DHA, dehydroepiandrosterone; PD, pregnanediol; PT, pregnanetriol; PTN, pregnantriolone; THA, tetrahydro-11-dehydrocorticosterone; THAldo, tetrahydro-aldosteron; THB, tetrahydro-corticosterone; THE, tetrahydrocortisone; THF, tetrahydrocortisol; THDOC, tetrahydro-11-deoxycorticosterone; THS, tetrahydro-11-deoxycortisol. The test was performed approximately 40 weeks after discontinuing osilodrostat. Abnormal metabolite concentrations are indicated in red font with an arrow. The background colors of the table correspond to the respective groups of steroid hormone metabolites, as explained in the accompanying legend.

**FIGURE 2:**
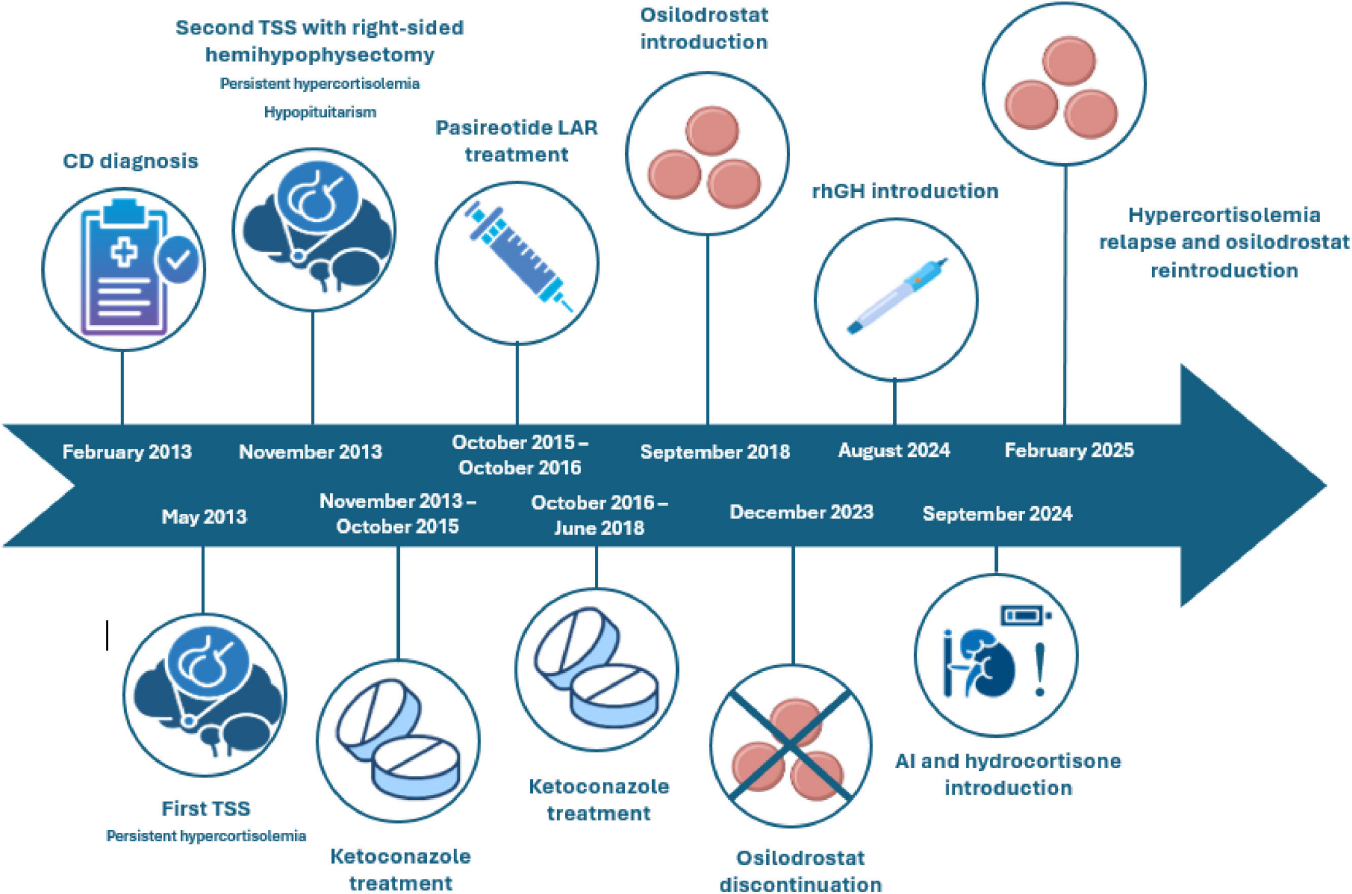
The figure summarizes the most relevant data of the presented patient since CD diagnosis in February 2013. AI, adrenal insufficiency; CD, Cushing’s disease; LAR, long-acting release; rhGH, recombinant human growth hormone; TSS, transsphenoidal surgery.

Figure 2 summarizes the timeline with the most relevant data of the presented patient.

## Discussion

In this paper, we present a unique case of a patient who experienced prolonged adrenocortical suppression following treatment with osilodrostat. Ultimately, after osilodrostat discontinuation, cortisol deficiency was unmasked upon initiation of rhGH therapy. During osilodrostat treatment, the patient experienced several episodes of hypocortisolism, which required dose reductions of osilodrostat and temporary administration of hydrocortisone. The first episode of AI occurred during the 20th week of therapy and was entirely unexpected, as biochemical and clinical assessments conducted in the 17th week had indicated that the patient had not yet achieved adequate disease control.

The data regarding the timing of AI during osilodrostat therapy are limited. In clinical trials, approximately 25% of patients experienced AI episodes, primarily during titration and adjustment of the osilodrostat dose. However, a nearly equal proportion of delayed AI has also been reported across studies (5, 6, 10). Real-world data, show that AI may occur at any point during osilodrostat therapy, even in patients maintained on a long-term stable dose (8, 9, 11). No specific risk factors have been clearly identified as predictors for the development of AI during osilodrostat treatment. Available evidence suggests that neither the administered dose of osilodrostat nor the initial severity of hypercortisolemia is consistently associated with the occurrence of AI (6, 8, 9, 11, 12). Instead, individual susceptibility to the drug appears to play a critical role in the onset of AI and prolonged adrenal suppression. As illustrated by the present case, AI may develop abruptly and unexpectedly. This highlights the importance of thoroughly educating patients on the risks of AI and its associated symptoms and providing them with hydrocortisone tablets for emergency use upon starting osilodrostat. Moreover, extending the dose-titration interval may be advisable in cases where a slower reduction in cortisol is needed to minimize the risk of hypocortisolism. It is noteworthy that the frequency of reported hypocortisolism episodes during the core phase of the study was higher in LINC-3 compared to LINC-4, which may be related to the shorter dose-escalation interval allowed during LINC-3 study (2 weeks vs. 3 weeks) (5, 10). Decisions regarding dose escalation should be guided by frequent cortisol monitoring, the observed rate of cortisol reduction, and the patient’s clinical response and tolerance to osilodrostat.

Over the following months of osilodrostat therapy, the patient experienced several additional episodes of AI, which required further tapering of the osilodrostat dose. The maintenance dose was eventually reduced to 1 mg in the evening every 3 days. This raises the question of whether an earlier attempt to discontinue osilodrostat should have been considered, and whether such a low dose could still provide any therapeutic benefit, given the known pharmacokinetics of the drug. However, considering individual susceptibility to osilodrostat, it cannot be entirely ruled out that even such a small dose may be sufficient for some patients. Notably, in the LINC-4 study, very low doses of osilodrostat (as low as 1 mg every other day) were allowed when clinically necessary (5). Additionally, at that time, limited data were available regarding the feasibility of complete osilodrostat discontinuation and the associated risk of hypercortisolemia recurrence.

In the presented patient osilodrostat was withdrawn in December 2023 after 270 weeks of treatment. We decided to closely monitor the patient for any signs of recurrence of hypercortisolemia, primarily through periodic LNSC measurements.

In August 2024, rhGH therapy was initiated due to severe GH deficiency (GHD). During follow-up 1 month later, the patient experienced another episode of AI, which required the initiation of hydrocortisone replacement therapy. The 11β-hydroxysteroid dehydrogenase type 1 (11βHSD1) enzyme, which converts cortisone to cortisol, is negatively regulated by GH. Thus, rhGH therapy - by normalizing 11βHSD1 activity - can increase cortisol metabolism and unmask an underlying cortisol deficiency (18, 19). Osilodrostat therapy in the presented patient appeared to more prominently inhibit adrenal steroidogenesis; however, the absence of overt cortisol deficiency was attributed to previously untreated GHD. The initiation of rhGH therapy subsequently unmasked persistent adrenal suppression related to osilodrostat.

Recently published reports have described eight other patients who experienced prolonged adrenal suppression following treatment with osilodrostat (12–16). This phenomenon was observed even in patients with intense hypercortisolism, indicating that the degree of hypercortisolism does not determine the occurrence of prolonged AI (12, 13, 16). This phenomenon has been observed in patients with various etiologies of CS, indicating that the type of the disease is not a determining factor either (12–16). The duration of osilodrostat exposure until discontinuation ranged from 10 weeks to 15 months and maximum daily osilodrostat dose ranged from 4 to 20 mg (12–14, 16). These reports indicate that both the duration of osilodrostat therapy and the administered dose appear to be unrelated to the risk of developing this complication. Metyrapone was administered prior to osilodrostat in three patients, potentially contributing to prolonged adrenal AI (12, 13). One patient (with nodular adrenocortical disease) received mifepristone followed by relacorilant and underwent cryoablation of the right adrenal gland prior to initiating osilodrostat treatment (15); the influence of cryoablation on the episode of prolonged adrenal suppression cannot be ruled out. It is noteworthy that in most reported cases, the first episode of AI led to osilodrostat discontinuation. However, in our patient, the occurrence of AI episodes prompted the initiation of temporary glucocorticoid replacement and a tapering of the osilodrostat dose instead. In fact, most AI episodes during osilodrostat treatment can be managed with dose adjustments rather than drug discontinuation. In two of the patients, osilodrostat also caused a persistent blockade of aldosterone synthesis, which required the initiation of mineralocorticoid replacement therapy (12, 13). Table 2 provides a summary of previously reported cases of osilodrostat-induced prolonged AI.

**TABLE 2:** Summary of published cases of prolonged adrenal insufficiency induced by osilodrostat.

Author	Patient characteristics (CS type, age, gender)	Hormonal tests before osilodrostat	Maximum osilodrostat dose (mg/day)	Time to first AI episode	Osilodrostat exposure until discontinuation	Time of prolonged AI
Poirier et al. (12)	CD, 51, F	n/a	4	6 months	6 months	6 weeks
Poirier et al. (12)	CD, 31, F	n/a	4	15 months	15 months	56 weeks
Poirier et al. (12)	ADCS, 41, M	mUFC: >30 × ULN	10	13 months	13 months	>39 weeks
Ferriere et al. (13)	EAS, 59, n/a	Serum cortisol: 3270 nmol/L ACTH: 440.4 pmol/L	20	1 week	24 weeks	52 weeks
Ferriere et al. (13)	CD, 51, F	mUFC: 9198.8 nmol/24 h (51.5 × ULN) ACTH: 34.35 pmol/L	10	10 weeks	10 weeks	>64 weeks
Tejani et al. (14)	CD, 41, F	mUFC: 63.4 nmol/24 h (0.46 × ULN) mLNSC: 5.05 nmol/L (2.0 × ULN) ACTH: 11.5 pmol/L	6	56 weeks	56 weeks	23 months
Velostki et al. (15)	AICS, 44, F	n/a	20	4 months	4 months	>3 years
Kaniuka-Jakubowska et al. (16)	EAS, 74, F	Serum cortisol: 1586 nmol/L mUFC: 6915.6 nmol/24 h (12.9 × ULN) ACTH: 50.2 pmol/L	4	4 weeks	15 months	>31 weeks
Presented case	CD, 41, M	Serum cortisol: 638 nmol/L mUFC: 580.0 nmol/24 h (4.2 × ULN) mLNSC: 11.9 nmol/L (4.7 × ULN) ACTH: 9.3 pmol/L	10	20 weeks	270 weeks	62 weeks

ACTH, adrenocorticotropin; ADCS, ACTH-dependent Cushing’s syndrome; AICS, ACTH-independent Cushing’s syndrome; CD, Cushing’s disease; CS, Cushing’s syndrome; F, female, M, male, mLSNC, mean late night salivary cortisol; mUFC, mean urinary free cortisol; ULN, upper limit of normal. Values were converted to consistent units (SI) for comparison.

The mechanism of action of osilodrostat, along with its relatively short half-life (4–5 h) (20), does not fully explain its potential to induce prolonged adrenal suppression – particularly after treatment discontinuation – even considering its strong inhibitory effect on 11β-OH (21). This suggests other, yet unknown long-term effects associated with this medication. It is possible that the cumulative dose of osilodrostat, rather than a direct dose-response relationship, plays a key role in this phenomenon. Furthermore, prolonged adrenal suppression may not be solely attributable to the enzymatic actions of osilodrostat. The potential adrenolytic effect of osilodrostat beyond direct enzymatic inhibition should also be considered. Notably, a recent report described adrenal gland shrinkage on computed tomography (CT) during osilodrostat therapy (22). Additionally, in the previously mentioned case of prolonged AI in a patient with adrenocortical disease, CT imaging after treatment with osilodrostat showed a decrease in the size of both adrenal glands; interestingly, imaging performed after cryoablation of the right adrenal gland showed no reduction in its size (15). Long-term effects of epigenetic modifications of steroidogenic enzymes in the pathogenesis of prolonged AI cannot be excluded either.

After 62 weeks following the cessation of osilodrostat therapy, the presented patient experienced a relapse of hypercortisolism. Osilodrostat was reintroduced at a low evening dose of 1 mg. Recent studies have shown that a single evening dose of osilodrostat is effective and can restore the circadian cortisol rhythm in patients with CD (23). The exact mechanism underlying the recovery of adrenal function, as well as the risk factors for the recurrence of hypercortisolism following an episode of prolonged AI, remain unclear. The recovery of adrenal function following prolonged suppression by osilodrostat may involve complex and heterogeneous mechanisms, including gradual restoration of the hypothalamic–pituitary–adrenal axis sensitivity, regeneration of adrenocortical cells or epigenetic modulation of steroidogenic enzyme expression. Nonetheless, this underscores the importance for regular reassessment of such patients, both for the recovery of adrenal function and for the early detection of hypercortisolism recurrence, with consideration given to timely reinitiation of osilodrostat therapy.

Similar to our findings, the authors of these reports observed that the patients exhibited low concentrations of DHEA-S despite elevated plasma ACTH levels, which suggests a potential inhibition of adrenal steroidogenesis upstream of 11β-OH. We conducted a steroid profile and confirmed significantly reduced excretion of DHEA metabolites. This finding suggests an additional mechanism of action for osilodrostat, potentially involving the inhibition of 17α-hydroxylase/17,20-lyase (17α-OH) activity and the synthesis of androgens through the delta-5 pathway. This effect appears to be evident only sometime after the initiation of osilodrostat treatment and has not been reported during short-term therapy during core phase of clinical trials. However, a progressive decline in DHEA-S levels was already observed during the LINC-3 study extension phase (24). In classical 17α-OH deficiency, the production of androstenedione is also markedly reduced or even absent. In the presented patient, no significant decrease in androstenedione concentration over the course of osilodrostat therapy was observed, in contrast to the marked reduction seen for DHEA-S. However, in the performed urinary steroid profile, androstenedione metabolites were found at the lower limit of the reference range. This allows for the hypothesis that osilodrostat may lead to a partial inhibition of 17α-OH activity. Notably, an *in vitro* inhibition of 17α-OH by osilodrostat has been reported (21). Despite the persistent adrenal suppression, the excretion of 11-deoxycortisol metabolites was within the reference range. Similar observation was made by Ferriere et al. - prolonged AI in presented patients was associated with low/normal serum 11-deoxycortisol concentrations (13). This indicates the need for further research on osilodrostat, especially to depict the full spectrum of its action and long-term consequences.

## Conclusion

We present a unique case of a patient with persistent CD who developed prolonged adrenal blockade during osilodrostat treatment, with cortisol deficiency ultimately unmasked following the initiation of rhGH therapy. The patient remained off osilodrostat for a total of 62 weeks. This case highlights how complex and unpredictable osilodrostat treatment can be and underscores the importance of close monitoring for both clinical and biochemical signs of AI. Additionally, we demonstrate that long-term use of osilodrostat likely does not selectively inhibit 11β-OH but may also impair the activity of 17α-OH. Given the increasing use of osilodrostat, further research is needed to elucidate the long-term consequences of this therapy and the mechanisms underlying prolonged adrenal suppression.

## Data Availability

The original contributions presented in this study are included in this article/supplementary material, further inquiries can be directed to the corresponding author.
